# Shallow wet irrigation reduces nitrogen leaching loss rate in paddy fields by microbial regulation and lowers rate of downward migration of leaching water: a ^15^N-tracer study

**DOI:** 10.3389/fpls.2024.1340336

**Published:** 2024-03-25

**Authors:** Tianyi Chen, Xiaoming Yang, Zheng Zuo, Huijuan Xu, Xingjian Yang, Xiangjian Zheng, Shuran He, Xin Wu, Xueming Lin, Yongtao Li, Zhen Zhang

**Affiliations:** ^1^ College of Natural Resources and Environment, Joint Institute for Environmental Research & Education, South China Agricultural University, Guangzhou, China; ^2^ College of Resources and Environment, Yunnan Agricultural University, Kunming, China

**Keywords:** shallow wet irrigation, soil nitrogen transformation, nitrogen leaching loss, ^15^N isotope tracer technique, microbial composition and function

## Abstract

China consumes 35% of the world’s fertilizer every year; however, most of the nitrogen fertilizers, which are essential for rice cultivation, are not used effectively. In this study, factors affecting the nitrogen leaching loss rate were studied in typical soil and rice varieties in South China. The effects of various irrigation measures on rice growth and nitrogen leaching loss were investigated by conducting experiments with eight groups. These groups included traditional irrigation (TI) and shallow wet irrigation (SWI). The TI is a common irrigation method for farmers in South China, maintaining a water layer of 5-8 cm depth. For SWI, after establishing a shallow water layer usually maintaining at 1-2 cm, paddy is irrigated when the field water level falls to a certain depth, then this process is then repeat as necessary. The nitrogen distribution characteristics were determined using ^15^N isotope tracing. In addition, the effects of nitrification, denitrification, and microbial composition on soil nitrogen transformation at different depths were studied by microbial functional gene quantification and high-throughput sequencing. The results revealed that in the SWI groups, the total nitrogen leaching loss rate reduced by 0.3-0.8% and the nitrogen use efficiency (NUE) increased by 2.18-4.43% compared with those in the TI groups. After the ^15^N-labeled nitrogen fertilizer was applied, the main pathways of nitrogen were found to be related to plant absorption and nitrogen residues. Furthermore, paddy soil ammonia-oxidizing archaea were more effective than ammonia-oxidizing bacteria for soil ammonia oxidation by SWI groups. The SWI measures increased the relative abundance of *Firmicutes* in paddy soil, enhancing the ability of rice to fix nitrogen to produce ammonium nitrogen, thus reducing the dependence of rice on chemical fertilizers. Moreover, SWI enhanced the relative abundance of *nirS* and *nosZ* genes within surface soil bacteria, thereby promoting denitrification in the surface soil of paddy fields. SWI also promoted ammonia oxidation and denitrification by increasing the abundance and activity of *Proteobacteria*, *Nitrospirae*, and *Bacteroidetes*. Collectively, SWI effectively reduced the nitrogen leaching loss rate and increase NUE.

## 1 Introduction

China, as one of the major rice-producing countries, accounts for one-fifth of the rice planting area and one-third of the rice production globally (Zhuang et al., 2019). Nitrogen fertilizers play a crucial role in rice cultivation. However, excessive irrigation can lead to low fertilizer use efficiency, and crops can only use 30%-40% of nitrogen fertilizers. About 50% of the applied nitrogen is lost to the environment due to ammonia volatilization, denitrification, runoff, and leaching. This situation has resulted in a range of serious environmental problems, such as soil acidification (Guo et al., 2010), groundwater pollution (Zhang et al., 2017), water eutrophication (Zhao et al., 2014, 2016), and air pollution (Wang et al., 2018; Cheng et al., 2021). Owing to the different water requirements of crops in each growth period, farmland irrigation water control can save water and improve crop quality and yield. The increasing studies related to the water-saving irrigation techniques have investigated grain yield, nitrogen use efficiency (NUE), denitrification, N_2_O and NO gas emissions derived from fertilizer and water regimes. Islam et al. (2018b) reported that the urea deep placement (UDP) increased grain yields by 13% during the Aman season. Qi et al. (2020) found that the water-saving irrigation techniques can increase rice yield by reducing total infiltration water. Some studies found that the combination alternate wetting and drying (AWD) irrigation and UDP drastically reduced N losses and increases NUE (Gaihre et al., 2015; Islam et al., 2018a, Islam et al., 2018b). Several studies have demonstrated that water-saving irrigation techniques can significantly decrease nitrogen emissions and leaching loss in paddy fields (Mao, 2002; Peng et al., 2011, 2012; Tan et al., 2013; Qi et al., 2020). Some studies reported that the combination AWD and UDP reduced ammonia volatilization and N_2_O emissions (Gaihre et al., 2015, 2018; Islam et al., 2018a). Shallow wet irrigation (SWI) is also a water-saving irrigation technology, and whether the use of SWI can reduce nitrogen loss and improve NUE. Additionally, nitrogen loss in paddy fields is influenced by microbial regulation. Therefore, it is necessary to understand the distribution, diversity, and abundance of microbial communities under SWI conditions, which can provide insights into microorganisms involved in nitrification in agricultural ecosystems.

Previous research indicated that nitrogen loss in paddy fields was regulated by soil microorganisms. Liu et al. (2020) found that irrigation greatly affected bacterial diversity. Das et al. (2016) reported that flooding might affect the composition and activity of rhizosphere microorganisms, consequently influencing the formation and accumulation of nitrogen forms in both rhizosphere soil and pore water. Soil aerobic conditions are important factors in determining the abundance of ammonia-oxidizing bacteria (AOB). Xu et al. (2020) found that dry-wet alternations increased soil oxygen content and further increased AOB abundance, which directly affected soil nitrification. Nitrification is the reaction of ammonia being converted into nitrite, which is in turn converted into nitrate by soil microorganisms; it is the dominant process of the soil nitrogen cycle. These processes of the nitrogen cycle are intricately connected to nitrogen loss. Compared with traditional irrigation methods, SWI treatment can provide better aerobic conditions for paddy soils. Whether SWI treatment can also increase the abundance of AOB, thereby reducing nitrogen loss in paddy fields. Meanwhile, understanding the response mechanisms of microbial communities to irrigation practices is vital for effectively preventing and controlling nitrogen loss.

In South China, the paddy growing season aligns with the summer rainy season, with an average annual precipitation of more than 1000 mm. Thus, runoff and leaching are the main processes of nitrogen loss (Chen et al., 2022). Currently, numerous studies have examined nitrogen runoff loss in paddy fields of South China (Ding et al., 2016; Issaka et al., 2019; Zeng et al., 2021). In our previous study, we found a 31.7% reduction in nitrogen loss from paddy field runoff in the SWI groups compared to that in the traditional irrigation (TI) groups (Zeng et al., 2021). However, limited studies are available on nitrogen leaching loss from paddy fields in South China. Paddy is a submerged crop, and leaching is one of the main ways of nitrogen loss. Owing to the limited number of studies in South China, quantifying the amount of leaching is difficult. Therefore, in the present study, we used a farmland underground leaching water collection device to conduct experiments with the following aims: (i) to monitor the effects of SWI measures on rice growth and nitrogen leaching loss; (ii) to analyze the mechanisms of nitrogen transport, distribution, and loss using ^15^N isotope tracing; and (iii) to analyze the effects of microbial colony structures and functional gene compositions on soil nitrogen transformation using molecular biotechnology. In this study, the leaching loss of nitrogen in paddy was quantified. We believe that this study provides a scientific basis and data to reduce the risk of nitrogen loss in paddy fields.

## 2 Materials and methods

### 2.1 Experimental site and soil characteristics

The experimental site was located at the South China Agricultural University, Guangzhou, Guangdong Province, China (23°15’N, 113°35’E). The South China Agricultural University is in the South China area. And this area is a densely populated and intensively farm region with a high cropping index (i.e., the average number of annual crop seasons). The experimental soil used was acid red soil, with a depth of 0 cm to 60 cm, which is a typical soil type in South China (Zeng et al., 2021). The topsoil had the following physicochemical characteristics: bulk density of 1.26 g·cm^-3^, soil pH of 5.83, soil organic matter of 15.49 g·kg^-1^, total phosphorus of 0.16 g·kg^-1^, and total nitrogen (TN) of 1.05 g·kg^-1^, with alkali-hydrolyzed nitrogen of 35.16 g·kg^-1^.

### 2.2 Experimental device

**Figure 1:**
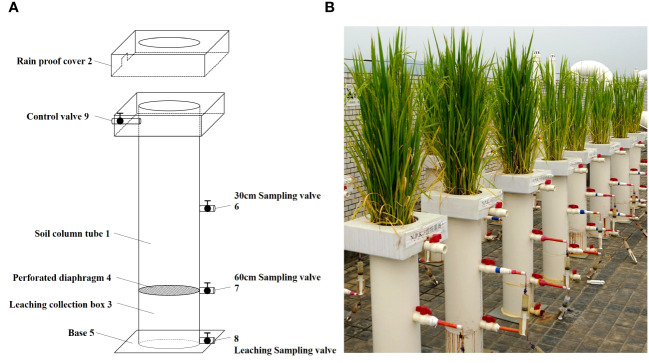
Schematic diagram of experimental device for collecting underground leaching water from farmland [**(A)** is design drawing and **(B)** is physical drawings].

The experimental device used to collect underground leaching water from the farmland included a soil column tube, rainproof cover, and leaching collection box (**Figure 1**). The experiment is a pot experiment and its device is made of 5mm thick PVC material. The specific size of the device has been given in **Supplementary Data** (**Supplementary Figure S1**). The rainproof cover was equipped with a through-hole corresponding to the soil column tube, and the lower end of the soil column tube was connected to the leaching collection box. There was a dense hole partition between the soil column tube and the leaching collection box. The bottom of the leaching collection box was connected to a base, and the side wall of the leaching collection box was equipped with leaching sampling valves. At least two depth sampling valves were installed on the side wall of the soil column tube. During sampling, the depth sampling valves at different depths were opened, and syringes were used to collect water samples at different depths of the leachate. The leaching sampling valve was opened, and a measuring cylinder was used to quantitatively collect the leaching water. During rice plant growth, flooding in the soil column tube was controlled by switching the field control valve to set different flooding depths and moisture patterns.

### 2.3 Experiment design

The experiment was set up with eight treatment groups according to two irrigation patterns (TI and SWI) and four nitrogen fertilizer application rates (0%, 40%, 70%, and 100%) with three replicates for each treatment group. The experimental fertilizers application rate was based on the recommended fertilizers application rate of Guangdong Province testing soil for formulated fertilization. The application rates of N, P and K fertilizers were 148, 67, 114 kg·ha^-1^, respectively. The treatment groups were set at 0, 40, 70, and 100 in accordance with 0%, 40%, 70%, and 100% of the TN fertilizer applied to the individual units, respectively. The urea concentration used was 10 atoms% ^15^N labeled urea. The amount of phosphate and potassium fertilizers applied was uniform for all the treatment groups. The specific experimental settings were shown in **Table 1**. The base fertilizer was applied to the rice seedlings before transplanting, the surface soil (0 cm -10 cm) was mixed thoroughly with minimal disturbance of the soil after fertilizer application. The tiller fertilizer was applied at the rice tillering stage to supply the required nutrients for late tillering and nodulation. The spike fertilizer was applied at the rice spike stage to supply nutrients required for flowering, fruiting, and fruit ripening. The tiller fertilizer and spike fertilizer were dissolved with 50 mL of pure water during application, and evenly applied to the surface soil. During the planting period, flooding in the installation was observed daily and supplemented according to the appropriate flooding conditions for SWI and TI. The water level of the TI treatment was supplemented to 8 cm when it fell below 5 cm, and a water level of 1 cm -2 cm was maintained for the SWI treatment.

**Table 1:** Experimental agricultural treatment design.

Treatment	Fertilizer application			Irrigation pattern
	N (kg·ha^-1^)	P (kg·ha^-1^)	K (kg·ha^-1^)	
TI ^a^-0	0	0	0	Conventional
TI-40	59	67	114	Conventional
TI-70	104	67	114	Conventional
TI-100	148	67	114	Conventional
SWI ^b^-0	0	0	0	Shallow-Wet
SWI-40	59	67	114	Shallow-Wet
SWI-70	104	67	114	Shallow-Wet
SWI-100	148	67	114	Shallow-Wet

a TI refers to traditional irrigation group.

b SWI refers to shallow wet irrigation.

### 2.4 Sample collection and analysis

#### 2.4.1 Water sample collection and analysis

The method of collecting and preserving leaching water samples is shown in **Supplementary Table S1**. The leaching water was sampled regularly every week, kept separately, and labeled according to sampling time and depth. The measured water data were recorded according to time.

The TN concentration of the collected samples was determined using a Unico UV-2800 (Unicoi Systems, Atlanta, GA, USA) spectrophotometer after performing potassium peroxodisulfate digestion. The concentrations of NH_4_^+^-N and ${\text{NO}}_{3-}-\text{N}$ were analyzed using a continuous-flow analyzer (Skalar, Breda, the Netherlands).


(1) $$V=\sum (V_{i})$$

where *V* denotes the leaching volume of a single device (L) and *V_i_* denotes the leaching volume of a single device in I sampling duration (L).


(2) $$V_{L}=\frac{V}{R_{t}\times R_{s}}\times 10^{-3}$$

where *V_L_* denotes the volume of leaching loss (t·hm^−2^·yr^−1^); *R_t_* refers to the planting time accounts for a proportion of the year, and *R_s_* denotes the proportion of area per hectare of a single device.


(3) $${NLV}_{i}=\frac{\sum C_{Ni}}{V}\times 10^{-3}$$

where *NLV_i_* represents the concentration of TN in leaching (g), and *C_Ni_* denotes the concentration of TN in leaching of a single device in I sample duration (mg·L^−1^).


(4) $$NLL=\frac{{NLV}_{1}-{NLV}_{2}}{W_{N}}\times 100$$

where *NLL* denotes the loss rate of TN through leaching (%); *NLV_i_* and NLV_0_ refer to *NLV* of N applied and N without applied, respectively, and *W_N_* denotes the amount of N applied.

#### 2.4.2 Soil and plant sample collection and analysis

The whole rice growth period was divided into three soil sampling periods as follows: before the application of the tiller fertilizer, before the application of the spike fertilizer, and before harvest. Soil samples were sampled at 3-5 points using soil sampling tubes and then mixed and bagged. The samples were stored separately according to sampling depth and were categorized as wet and dry. Soil samples that needed to be air-dried were stored in a cool and ventilated place after natural air-drying, whereas fresh soil was stored at -80°C. Plant samples were collected only at harvest time. Further, plant samples from each device were cut flush, measured for wet weight, dried in a 70°C oven, cooled, weighed for dry weight, and finally crushed, ground, and stored in bags.

The TN content of soil was analyzed using the Kjeldahl method, and the nitrogen contents of both the soil and plant samples were measured using an elemental analyzer (Vario MICRO cube, Elementar, Germany). The alkali-hydrolyzed nitrogen content of soil was analyzed using the alkaline hydrolysis method. The NH_4_^+^-N and ${\text{NO}}_{3-}-\text{N}$ concentrations were analyzed using a continuous-flow analyzer (Skalar, Breda, the Netherlands) (Wang et al., 2021). The nitrogen content of the plant samples was measured using an elemental analyzer (Vario MICRO cube, Elementar, Germany).

#### 2.4.3 Isotope abundance determination

The atom% ^15^N abundance was analyzed using a stable isotope mass spectrometer (IsoPrime 100, Elementar, Germany). The proportion of the isotopes to the fertilizer (Ndff) was calculated based on the natural abundance of isotopes, and the Plant ^15^N use efficiency, Soil ^15^N residue rate, ^15^N leaching loss rate are calculated with reference to the method of Wang et al. (2017) and Li et al. (2018). The background of ^15^N abundance in soil was 0.368 atom%. *Ndff* was calculated as follows:


(5) $$Ndff(\%)=\frac{a-b}{c-d}\times 100$$

where *a* is atom% ^15^N abundance in the plant/soil/water samples, *b* is atom% ^15^N abundance in the control plant/soil/water samples, *c* is atom% ^15^N abundance of the fertilizer, and *d* is natural atom% ^15^N abundance (0.368 atom% ^15^N).

Plant ^15^N use efficiency was calculated as follows:


(6) $$\text{Plant}{\;}^{\mathrm{15}}\text{N use efficiency }(\%)\text{ =}\frac{Ndff\times e_{p}\;\times W_{p}}{f}\times 100$$

where *e_p_* is the ^15^N concentration of the plant (%), *W_p_* is plant dry weight (g), and *f* is the amount of fertilizer (g).

Soil ^15^N residue rate was calculated as follows:


(7) $$\text{Soil}{\;}^{\mathrm{15}}\text{N residue rate }(\%)\text{ =}\frac{Ndff\times {\;e}_{s}\;\times W_{s}}{f}\times 100$$

where *e_s_* is the ^15^N concentration of plant (%), and *W_s_* is the soil dry weight (g).

^15^N leaching loss was calculated as follows:


(8) $${\;}^{\mathrm{15}}\text{N}\;\text{leaching loss rate }\;(\%)\;=\frac{Ndff\times {\;e}_{L}\;\times {\;W}_{L}}{f}\times 100$$

where *e_L_* is the ^15^N concentration of leaching (%), and *W_L_* is the leaching weight (g).

#### 2.4.4 High-throughput sequencing and functional gene quantification of soil microorganisms

The genomic DNA was extracted from the soil using the FastDNA^®^ SPIN Kit for Soil (MP Biomedicals, CA, USA). The concentration of DNA was measured using a NanoDrop2000 spectrophotometer. Polymerase chain reaction (PCR) of the rRNA gene was conducted using the universal 16S rRNA primers (338F: ACTCCTACGGGAGGCAGCAG and 806R: GGACTACHVGGGTWTCTAAT). The 16s raw data were deposited in the National Center for Biotechnology Information (NCBI) Sequence Reads Archive (SRA) (accession number: PRJNA1087108). The resulting product was detected using 2% agarose gel electrophoresis. The PCR product was purified and quantified using the Quantus™ Fluorometer and AxyPrep DNA Gel Extraction Kit, respectively. The DNA library was built using the NEXTFLEX Rapid DNA-Seq Kit and sequenced using the Miseq PE300 platform (Illumina, California, USA). The original sequencing sequence was subjected to quality control using Trimmomatic software. The sequences were clustered into operational taxonomic units (OTU) using UPARSE software (version 7.1 http://drive5.com/uparse/) based on 97% similarity. Chimeras were removed from the dataset using UCHIME software. Species classification annotations were assigned to each sequence using RDP classifiers. The alignment threshold was set to 70% based on the Silva database (SSU128).

Functional gene quantitative primer information and amplification system conditions for bacteria and archaea are shown in **Supplementary Tables S2** and **S3**. Genomic DNA was extracted using the Magnetic Bead Method Soil and Fecal Genomic DNA Extraction Kit. After the genomic DNA was photographed in a gel imaging analyzer using Beijing Liuyi DYY-6C at a concentration of 1%, a voltage of 120 V, and an electrophoresis time of 20 min, the DNA concentration and purity were detected using Thermo NANo DROP8000. The standard quality granules were provided by Wuhan Tianyi Huiyuan Biotechnology Co., Ltd. and diluted 10-fold to obtain six concentration gradients of 1E9, 1E8, 1E7, 1E6, 1E5, 1E4, and 1E3, and the reactions were prepared using qPCR96Well and SYBR^®^ Select Master Mix (2X) kits, with each sample analyzed in triplicate.

The samples were quantified on an Applied Biosystems StepOnePlusTM Real-Time System and Bio-Rad CFX96 Real-Time System, and the same primers and conditions were applied according to the standard curve for quantitative PCR 3-well detection. A 96-well plate with standard mass pellets was used as a positive control for error correction, and no template control (NTC)was used as a negative control. The test results were analyzed usin StepOne v2.3 and BioRadCFXManager software. Refer to the research of Liu et al. (2022) and Man et al. (2022), the relative abundance of functional genes was calculated as:


(9) $$\text{Relative abundance of bacteria }amoA\text{ genes}=\frac{\text{Bacterial }amoA\text{ gene copy numbers}}{\text{Bacterial }16\text{S gene copy numbers}}$$

(10) $$\text{Relative abundance of archaeal }amoA\text{ genes}=\frac{\text{Archaeal }amoA\text{ gene copy numbers}}{\text{Archaeal }16\text{S gene copy numbers}}$$

(11) $$\text{Relative abundance of bacteria }nirS\text{ genes}=\frac{\text{Bacterial }nirS\text{ gene copy numbers}}{\text{Bacterial }16\text{S gene copy numbers}}$$

(12) $$\text{Relative abundance of bacteria }nosZ\text{ genes}=\frac{\text{Archaeal }nosZ\text{ gene copy numbers}}{\text{Bacterial }16\text{S gene copy numbers}}$$

#### 2.4.5 Statistical analysis

All experiments were conducted in triplicate, and the data were presented as the arithmetic mean values. Statistical analysis was conducted using SPSS statistics version 20.0. The experimental data were plotted using Origin 2022 software. In each case, the data were statistically analyzed using a one-way analysis of variance, with the minimum level of significance set at p< 0.05.

## 3 Results

### 3.1 Physicochemical properties of soil and rice plants

**Figure 2:**
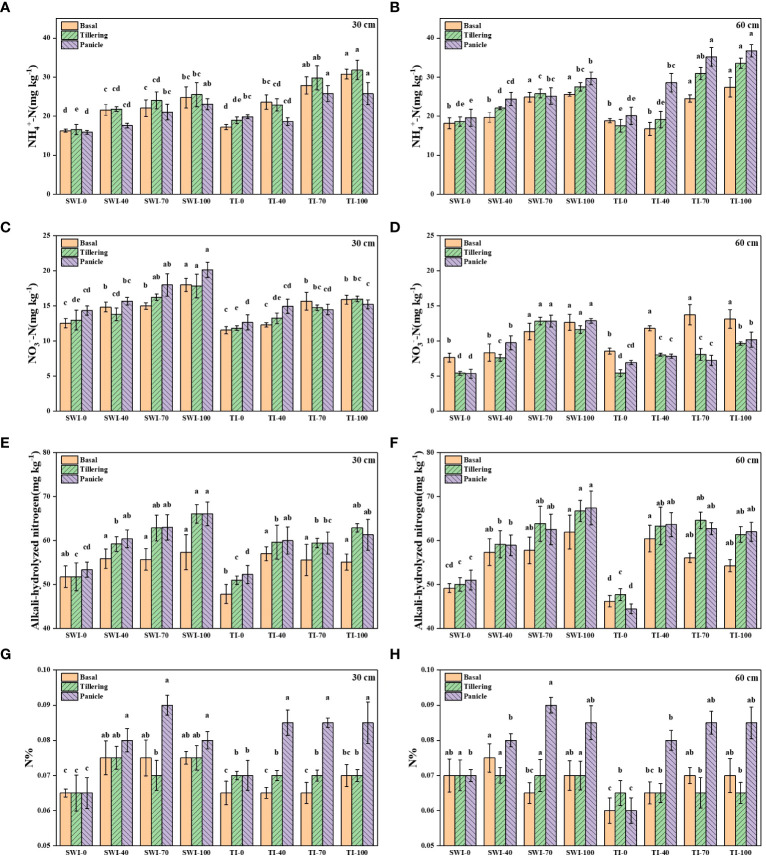
The contents of NH_4_^+^-N **(A, B)**, NO_3_^-^-N **(C, D)**, alkali-hydrolyzed nitrogen **(E, F)** and nitrogen contents **(G, H)** in 0–30 cm and 30–60 cm soil layers of rice under different treatments. Means within the same item followed by different letters are significantly different (LSD, p < 0.05).

The changes of soil nitrogen content at different depths of rice under different treatments were shown in **Figure 2**. The ammonium nitrogen content under TI treatment was significantly higher than that under SWI treatment at different growth stages and depths (p<0.05, **Figures 2A, B**). The changes of soil ammonium nitrogen content in 30-60 cm depth soil layers of paddy under different irrigation measures were similar to those in 0-30 cm depth. Overall, nitrate nitrogen content is higher in 0-30 cm depth soil layers than in 30-60 cm depth. During the same fertilization period, the nitrate nitrogen content of the SWI treatment was higher than that of the TI treatment (p<0.05, **Figures 2C, D**). The alkali-hydrolyzed nitrogen content of SWI treatment was significantly higher than that of TI treatment (p<0.05, **Figures 2E, F**). Under two different soil depths, with the increase of N fertilizer application in the treatment group, the contents of ammonia nitrogen, nitrate nitrogen and alkali-hydrolyzed nitrogen in soil will also increase. The soil nitrogen content after basal and tillering in the treatments of SWI-40, SWI-70, and SWI-100 were significantly higher than that of other treatments(p<0.05). The peak value of soil nitrogen content was SWI-70 treatment in 0-30 cm depth soil (0.09%) during panicle (**Figures 2G, H**).

The physicochemical properties of rice plants under different treatments were shown in **Table 2**. The growth differences of paddy in different treatment groups were shown in **Supplementary Figure S2**. Compared with TI treatment, SWI treatment could increase rice yield, seed setting rate and plant nitrogen content. The nitrogen content of plants was significantly increased with high nitrogen fertilizer application of SWI-70 and SWI-100 (p<0.05).

**Table 2:** Physicochemical properties of rice plants under different treatments.

Treatment	Plant height (cm)	Grain yield (g)	Straw yield (g)	Seed-setting rate (%)	Nitrogen content (%)
TI ^a^-0	86.91 ± 1.81c	6.21 ± 0.94d	19.06 ± 1.50d	65.50 ± 1.50de	0.92 ± 0.03c
TI-40	91.02 ± 1.88bc	15.37 ± 2.77cd	46.17 ± 4.96c	70.00 ± 1.75d	0.96 ± 0.04c
TI-70	102.61 ± 2.44ab	42.91 ± 5.84b	67.76 ± 3.41b	81.50 ± 1.40c	1.02 ± 0.08bc
TI-100	104.38 ± 5.64a	75.61 ± 7.18a	88.06 ± 5.23a	85.00 ± 1.50ab	1.16 ± 0.02a
SWI ^b^-0	91.34 ± 2.85bc	5.68 ± 0.34d	19.34 ± 0.66d	64.00 ± 1.00e	0.95 ± 0.01c
SWI-40	93.85 ± 2.44b	18.64 ± 2.67c	45.52 ± 2.04c	68.00 ± 3.25de	1.22 ± 0.09a
SWI-70	101.14 ± 3.61ab	45.15 ± 4.85b	68.77 ± 5.41b	82.25 ± 2.50bc	1.11 ± 0.01ab
SWI-100	105.61 ± 3.11a	76.41 ± 5.81a	94.45 ± 8.75a	88.00 ± 0.75a	1.14 ± 0.00a

a TI refers to traditional irrigation group.

b SWI refers to shallow wet irrigation group.

Means (n = 3) within a column followed by different letters are significantly different (LSD, p < 0.05).

### 3.2 Leaching loss concentration and loss volume

**Figure 3:**
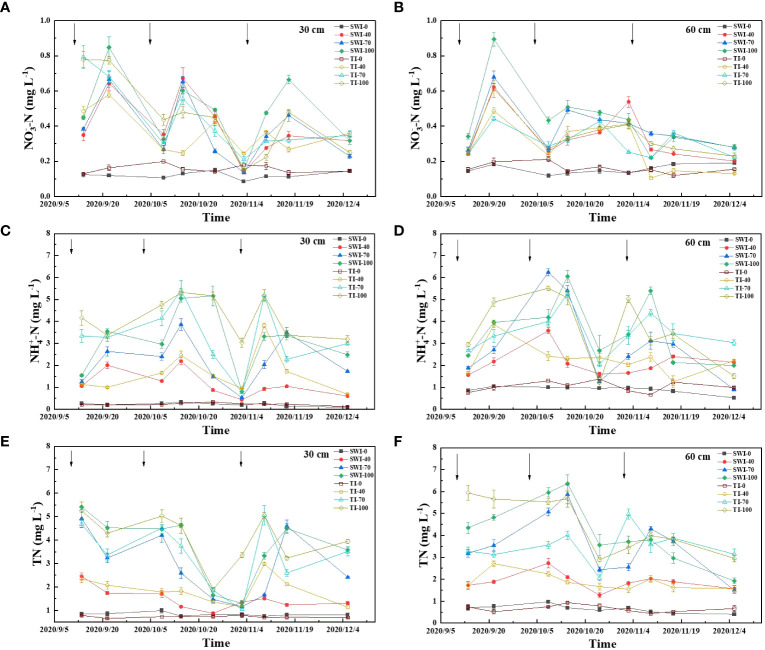
Dynamics of leached NO_3_^-^-N **(A, B)**, NH_4_^+^-N **(C, D)** and TN **(E, F)** concentrations in rice under different water fertilizer management practices (Arrows represent fertilization).

The dynamic changes of rice leaching nitrogen concentration under different irrigation measures were shown in **Figure 3**. The increase of fertilization rate can increase the concentration of leaching total nitrogen loss. The SW-100 and TI-100 treatment had the highest concentrations of leached total nitrogen, which were 1.30-5.41 mg·L^-1^ and 1.89-5.25 mg·L^-1^. The effect of each fertilization on the total nitrogen concentration in 30-60 cm depth soil layers was less abrupt than in 0-30 cm depth, and the concentration of total nitrogen increased first and then decreased slowly with the growth of rice (**Figures 3A, B**).

The concentration of leached ammonium nitrogen was similar to that of total nitrogen, and the main form of soil nitrogen leaching was ammonium nitrogen (**Figures 3C, D**). The leaching ammonium nitrogen concentration responded quickly to the three nitrogen fertilizer applications and had a large range. The TI-100 and TI-70 were the most obvious treatments with concentrations ranging from 3.04-5.32 mg·L^-1^ and 0.76-5.42 mg·L^-1^. The content of leaching nitrate in each treatment was significantly lower than that of ammonium nitrogen, and the content of leaching nitrate in each treatment fluctuatingly decreased with the growth period (**Figures 3E, F**).

**Figure 4:**
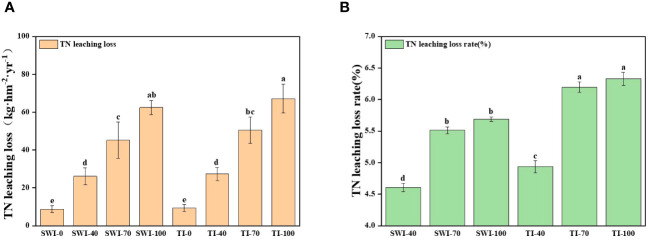
Changes in the amount **(A)** and rate **(B)** of total nitrogen loss from rice leaching under different treatments.

The changes of total nitrogen loss in leaching (Equation 3) of rice under different treatments are shown in **Figure 4**. At the same time, the leaching loss volume of SWI treatment was significantly lower than that of TI treatment (Equations 1, 2; p<0.05, **Supplementary Figure S3**). The total nitrogen leaching loss rate (Equation 4) of each treatment was 4.60-6.32%, and the TI-100 and TI-70 were the highest treatments. Compared with the TI treatment, although the total leaching nitrogen concentration of SWI treatment was slightly higher than that of TI treatment, the total nitrogen leaching loss of SWI treatment was significantly lower. Therefore, the total nitrogen loss rate of SWI treatment was low, indicating that SWI treatment could effectively reduce nitrogen loss.

### 3.3 Distribution of ^15^N in the soil-plant-water system after fertilization

**Figure 5:**
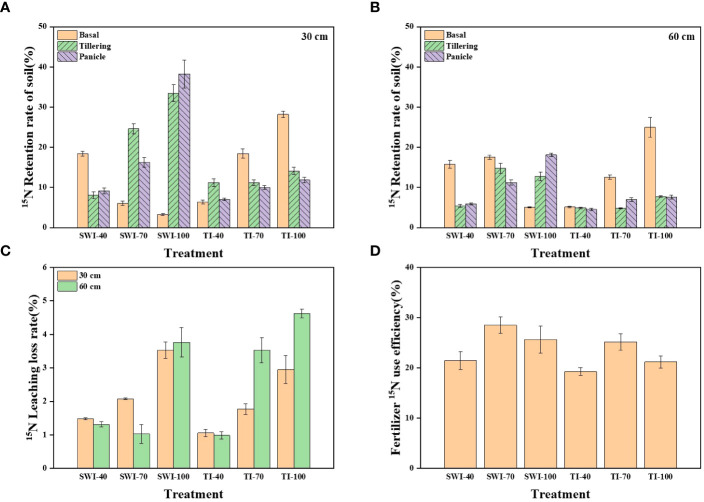
**(A)** Residual rate of ^15^N-labeled nitrogen fertilizer in rice 0-30 cm soil layer under different treatments; **(B)** Residual rate of ^15^N-labeled nitrogen fertilizer in 30-60 cm soil layer; **(C)** ^15^N leaching loss rate of rice nitrogen fertilizer under different treatments; **(D)** NUE of ^15^N-labeled nitrogen fertilizer under different treatments.

The residue and leaching loss rate of ^15^N labeled nitrogen fertilizer in rice soil under different treatments is shown in **Figure 5**. After the basal, the soil nitrogen residue rate of SWI treatment decreased with the increase of fertilizer rate, while that of TI was the opposite. After the tillering and panicle, the nitrogen residue rate of SWI treatment (Equations 5, 7) in 0-30 cm depth soil layers was increased (**Figure 5A**). After the panicle, the SWI-100 was the treatment with the highest residue rate, reaching 38.25%. Analyzing the soil nitrogen residue rate in two depth soil layers, it was found that nitrogen fertilizer in the SWI treatment was more likely to accumulate in 0-30 cm depth soil layers, while nitrogen fertilizer in the TI treatment was similar at two depths soil layers (**Figures 5A, B**).

On the whole, the leaching loss rate of nitrogen fertilizer in 30-60 cm depth soil layers (1.06-4.63%) under TI treatment was higher than that in 0-30 cm depth (0.99-2.95%). Increasing the nitrogen fertilizer application rate has a tendency to widen the gap between the leaching loss rate (Equations 5, 8) of nitrogen fertilizer in the two depths. However, compared with TI treatment, the difference in the leaching loss rate of nitrogen fertilizer in the SWI treatment was lower between the two depths (**Figure 5C**). From **Figure 5D**, it can be seen that the fertilizer NUE (Equations 5, 6) of each treatment ranged from 19.28-28.50%, with the peak value of SWI-70 treatment. Under different irrigation measures, the fertilizer NUE with 70% nitrogen application rate was the highest. Compared with TI treatment, the SWI treatment could increase the NUE by 2.18-4.43% under the same nitrogen application rate.

### 3.4 Changes in soil microbial communities

The diversity index analysis of soil bacterial communities in each treatment was shown in **Supplementary Table S4**. The Chao1 and ACE indices of the 0-30 cm depth soil in the SWI groups were greater than those of the 30-60 cm depth soil, indicating that the microbial community richness of the 0-30 cm depth soil in the SWI groups was higher than that of the 30-60 cm depth soil. The Shannon index of the SWI groups was generally smaller than that of the TI groups, and the Shannon index of the 0-30 cm depth soil was generally smaller than that of the 30-60 cm depth soil. The Simpson index was higher in the SW-0 group compared to that in the TI-0 group, whereas the other SW groups showed lower Simpson indices than the TI groups. Therefore, the SWI measures enhanced both the homogeneity of soil microorganisms and the diversity of soil microbial communities.

**Figure 6:**
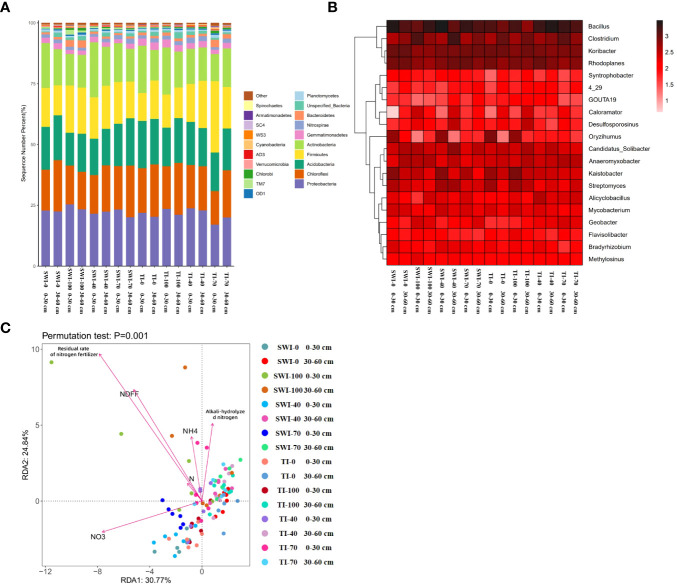
**(A)** Relative abundance composition of bacterial communities in each treatment group based on phylum levels; **(B)** Relative abundance composition of bacterial communities in each treatment group based on genus level; **(C)** Redundancy analysis (RDA) of NH_4_^+^-N, ${\text{NO}}_{3-}-\text{N}$ , alkali-hydrolyzed nitrogen, nitrogen content (N%), Ndff and nitrogen fertilizer residual rate under different irrigation and fertilization treatment conditions.

High-throughput sequencing of soil microorganisms was used to analyze the microbial community at the phylum level. (**Figure 6A**). In the relative abundance of *Proteobacteria*, the vast majority of the 0-30 cm depth soil was higher than that in the 30-60 cm depth soil, with the SWI-100 groups demonstrating the highest relative abundance (25.47%). The SWI groups exhibited a higher relative abundance of *Firmicutes* than the TI groups. Changes in the microbial community at the genus level are shown in **Figure 6B**. In the SWI-0 and SWI-100 groups, the relative abundance of *Bacillus*, *Clostridium*, and *Anaeromyxobacter* was higher compared to those in the TI-0 and TI-100 groups.

The redundancy analysis (RDA) revealed (**Figure 6C**) that the differences between the bacterial communities of soils at the two depths in the SWI groups were greater. At the 0-30 cm depth, the bacterial communities showed a positive correlation with the soil nitrate content and a negative correlation with the soil alkaline and ammonium nitrogen content, while the opposite trend was observed at the 30-60 cm depth. This indicated that the form of nitrogen in the soil at different depths were related to soil microbial communities. Differences in soil microbial communities at the 0-30 cm depth were closely related to nitrogen forms. This indicated that the fate of nitrogen in the paddy soil was closely linked to the microbial communities in the surface soil. The SWI measures could promote this phenomenon.

### 3.5 Changes in the soil microbial functional gene abundance

**Figure 7:**
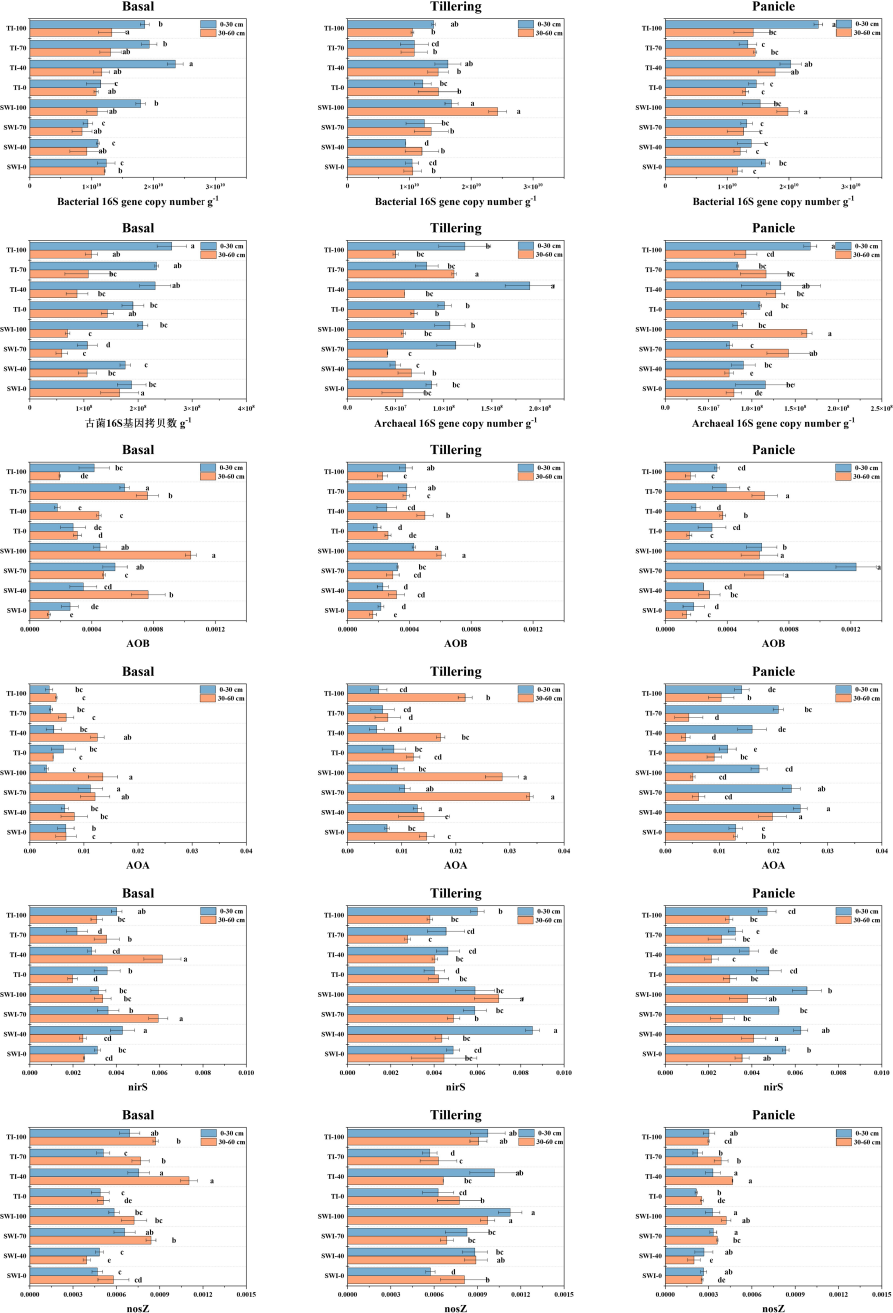
Changes in the copy number of soil bacteria 16S gene, copy number of soil archaea 16S gene, relative abundance of soil bacteria amoA gene, relative abundance of soil archaea amoA gene, relative abundance of soil bacteria *nirS* and *nosZ* gene at 0-30 cm and 30-60 cm soil in three reproductive periods.

**Figure 7** shows the results of soil microbial functional gene abundance determination. The 16S gene copy numbers of soil bacteria and archaea at the 0-30 cm depth were larger than those at the 30-60 cm depth. The 16S gene copy numbers of soil bacteria were 1-2 orders of magnitude larger than that of archaea. This indicated that the surface or rhizosphere soil exhibits the most concentrated distribution of soil microorganisms. The analysis of the abundance of soil ammonia-oxidizing microbial functional genes showed that the relative abundance of the bacterial *amoA* gene in the SWI groups was significantly higher than that in the TI groups (Equation 9). After fertilization with the tiller fertilizer, the relative abundances of the *amoA* gene in the 0-30 cm and 30-60 cm depth soils were 4.30 × 10^-4^ and 6.07 × 10^-4^, respectively, in the SWI-100 groups. After the fertilization of the panicle fertilizer, the relative abundance of the soil bacterial *amoA* gene in the SWI groups was significantly higher compared to the TI groups. The relative abundance of soil archaea genes (Equation 10) was approximately ten times higher compared to that of the bacterial *amoA* gene.

The analysis of soil denitrifying bacterial functional genes showed that there was a higher relative abundance of the soil bacterial *nirS* gene in the SWI groups than that in the TI groups (Equation 11). After each fertilization, the relative abundance of the soil bacterial *nirS* gene in the SWI groups increased, whereas that in the TI groups decreased during the later stage of rice growth. The SWI measure resulted in an increase in the relative abundance of the bacterial *nirS* gene both in the surface soil and in the deep soil during the middle stage of rice growth in paddy fields.

## 4 Discussion

### 4.1 Changes in physical and chemical properties of soils and rice plants

In this study, the SWI treatment increased the NO_3_^-^-N content and decreased the NH_4_^+^-N content of the paddy soil. These findings align with the research conducted by Wang and Huang (2021) on nitrogen content in paddy soils under water-saving irrigation practices. Soil ammonium nitrogen and nitrate nitrogen tended to migrate to deep soil; however, the migration rate of ammonium nitrogen was slower than that of nitrate nitrogen. Soil ammonium nitrogen was prone to adsorption onto soil colloidal particles, leading to its slower migration, whereas ammonium nitrogen converted into nitrate nitrogen was easier to move with water (Wang et al., 2019). After the application of nitrogen fertilizer, SWI measure can slightly increase the content of alkali-hydrolyzable nitrogen and soil nitrogen in paddy soil, indicating that reducing irrigation water will increase the content of soil organic matter and alkali-hydrolyzable nitrogen (Ma, 2018), and the increase is positively correlated with the amount of fertilizer applied.

The SWI treatment and increasing nitrogen fertilizer application rate could increase rice yield, seed setting rate and nitrogen content, but had little effect on the increase of rice plant height. This is similar to the research of Islam et al. (2016) that studying the alternate wetting and drying irrigation increased grain yield by 16%. The leaf size, tillering number and heading status of rice with 70% nitrogen application rate were significantly better than those with 40% nitrogen application rate. Postponing and reducing the application of nitrogen fertilizer can reduce the rate of nitrogen transfer in leaves, delay leaf senescence, and lead to high nitrogen accumulation in rice (Ye et al., 2013).

### 4.2 Dynamic changes in leaching loss concentration and loss volume

The leaching TN concentration of the SWI-100 treatment groups was found to be higher compared to that of the TI-100 treatment groups, which might be because of less water input and less dilution of nitrogen under SWI. The leaching NH_4_^+^-N concentration of the SWI-100 treatment groups was lower than that of the TI-100 treatment groups, which might be because of the reduction of the surface soil under TI. This hindered the nitrification of NH_4_^+^-N, and the applied urea was more easily converted and accumulated into NH_4_^+^-N (Valerie et al., 2023). Simultaneously, there was a posterior shift in the peak concentration of leaching NH_4_^+^-N in the SWI treatment groups. NH_4_^+^-N may be easily adsorbed by soil colloids, resulting in a slower rate of migration. Moreover, the leaching effect of the SWI treatment was weaker than that of the TI treatment, resulting in a slower downward migration of NH_4_^+^-N with gravity water. The predominant form of nitrogen leaching loss in paddy fields was NH_4_^+^-N, with a significantly higher concentration than NO_3_^-^-N. This was consistent with the findings of Ji et al. (2011), which indicated that NH_4_^+^-N was the primary form of nitrogen leaching loss, constituting 39.70% of the TN loss. Root uptake is the main way for rice to absorb nitrogen (Yang et al., 2023). Zhang et al. (2016) found that rice roots had a strong preference for NH_4_^+^-N absorption. Supplying NH_4_^+^-N fertilizers to ammonium-loving crops can improve the NUE of paddy and supplying both NH_4_^+^-N and NO_3_^-^-N can promote nitrogen absorption and root growth (Dong et al., 2023). Therefore, controlling moisture in paddy fields can slow down the rate of nitrogen leaching. Simultaneously, fertilizer-converted nitrogen remained in the soil of rice roots for a longer time during downward migration, promoting more forms of nitrogen to be absorbed by the plants and thus limiting nitrogen leaching losses.

The TN leaching losses in the SWI-100 and TI-100 treatment groups were 62.45 and 67.21 kg·hm^-2^·yr^-1^, respectively. Compared with the TI treatment groups, the SWI groups could reduce leaching water in the paddy field. This is similar to the study of Qi et al. (2020), which showed that dry-wet alternations significantly reduced leaching water by 21.90%. SWI reduced the amount of leaching loss by 3.0-15%, thereby reducing the TN leaching loss by 5.0-11% and the TN leaching loss rate by 0.30-0.80%. Therefore, SWI may be an effective method to reduce nitrogen leaching in paddy fields.

### 4.3 Distribution of ^15^N in the soil-plant-water system after fertilization

**Figure 8:**
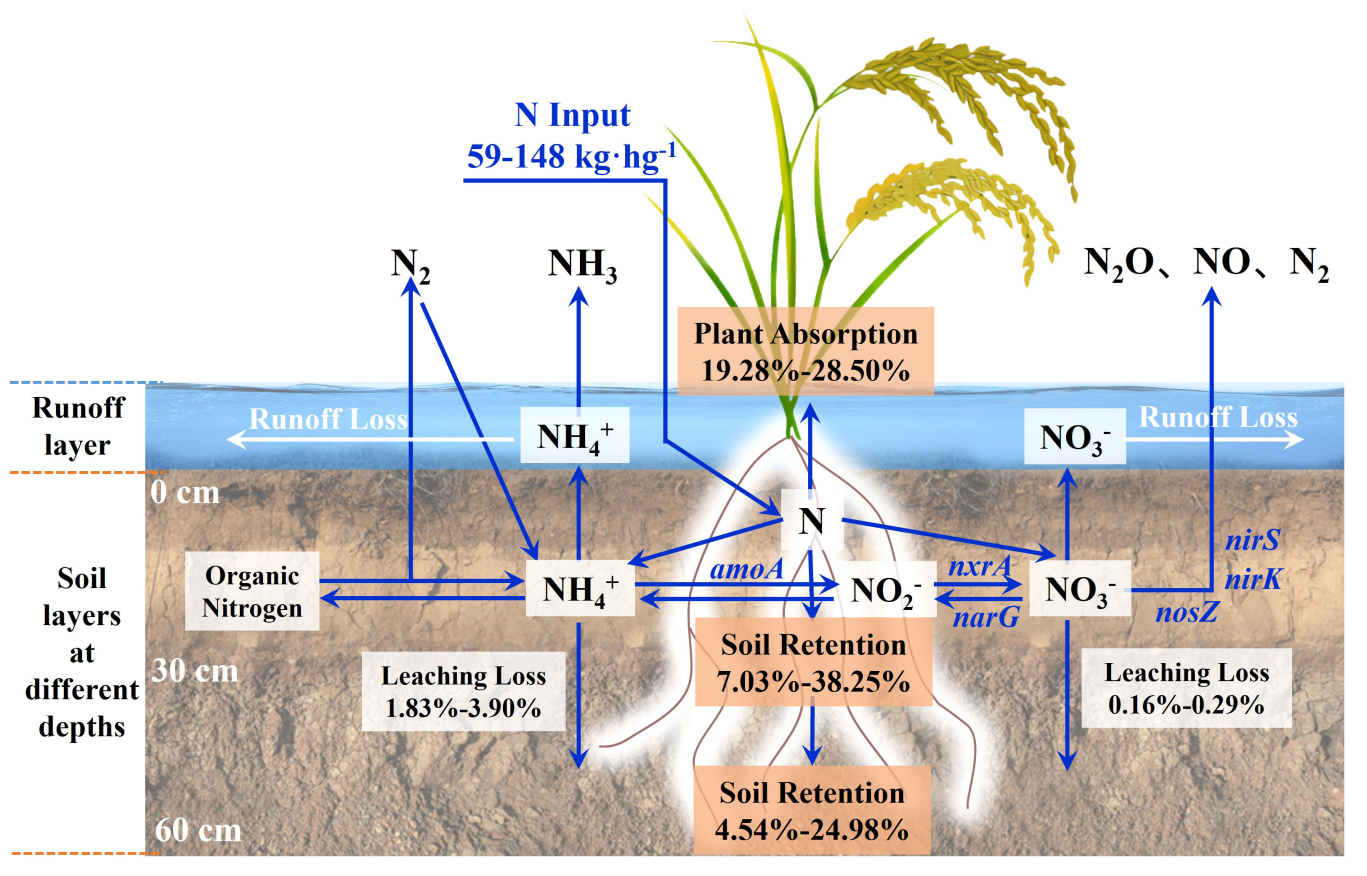
Nitrogen fate of ^15^N-labeled nitrogen fertilizer.

**Figure 8** shows the nitrogen flow trend of paddy fields with ^15^N nitrogen fertilizer. Plant uptake (19.28-28.50%) and soil nitrogen residue were the main destinations of nitrogen fertilizer. SWI treatment could increase NUE by 2.18-4.43% under the same nitrogen application rate. Under the same irrigation measures, the NUE of 70% nitrogen application rate was the highest, which was 28.50%. Too high or too low nitrogen application rate would reduce the NUE, which was similar to the result that Zhang et al. (2012) found that the ^15^N-labeled NUE of rice plants was 26-30%. The accumulation of nitrogen fertilizer in soil under SWI treatment mainly occurred in the middle and late stages of rice growth (after the application of tillering or panicle), while TI treatment mainly occurred in the early stage of rice growth (after the application of basal), which is consistent with previous studies. Li et al. (2018) found that 10.30-36.40% of the basal remained in paddy soil. The leaching loss of nitrogen in various forms accounted for only 1.31-4.63% of nitrogen fertilizer. This was similar to that of many previous studies, such as the studies of Ji et al. (2011); Peng et al. (2011); Zhang et al. (2017); Han et al. (2021), and their leaching loss rates of nitrogen fertilizer were 3.50-5.40%, 3.50-5.40%, 1.40-6.40% and 0.66-2.28%, respectively. It was due to the high rainfall, low soil organic matter content and large surface runoff in South China (Zeng et al., 2021), which leads to the low rate of nitrogen fertilizer leaching. With the increase of nitrogen fertilizer application, the nitrogen leaching loss rate also showed an increasing trend, which was similar to the previous study (Shen et al., 2022 *;* Zhang et al., 2021). This is also similar to the result of total nitrogen leaching loss in Section 3.2, indicating that appropriately reducing the nitrogen application rate in agricultural production can effectively reduce the risk of nitrogen leaching loss in paddy fields. Nitrogen fertilizer from SWI treatment is more likely to accumulate in 0-30 cm depth soil layers, which is the same as the finding of Zhang et al. (2012). Increasing the fertilization rate could significantly increase the nitrogen residue rate in 0-30 cm depth soil layers under SWI treatment and in 30-60 cm depth soil layers under TI treatment. The difference verifies that SWI treatment can effectively reduce soil nitrogen leaching loss in paddy fields.

### 4.4 Effects of different fertilization measures on soil bacterial community composition

The SWI measures enhanced both the homogeneity of soil microorganisms and the diversity of soil microbial communities (**Supplementary Table S4**). The SWI measures could increase the relative abundance of *Proteobacteria* in the surface soil. Kowalchuk and Stephen (2001) found that the first step of the nitrification process, that is, the oxidation of ammonia to nitrite was the limiting step in the cycle. This stage was usually carried out by AOB of *β-Proteobacteria* and *γ-Proteobacteria* (Schleper et al., 2005). The SWI increased the relative abundance of *Firmicutes* in the paddy soil. Additionally, *Firmicutes* were found to have the ability to fix nitrogen and produce a large amount of ammonium nitrogen during rice growth (Wang et al., 2017). This may complement the relatively low NH_4_^+^-N content in the paddy soil and water under SWI measures, reducing the dependence of rice on chemical fertilizers, and thereby increasing the NUE. Among them, Mevel and Prieur (2000) found that *Bacillus* could improve heterotrophic nitrification activity under aerobic conditions. Simultaneously, *Clostridium* and *Anaeromyxobacter* could promote nitrogen fixation under anaerobic conditions (Masuda et al., 2020; Han et al., 2021). The relative abundance of *Nitrospirae* in 0-30 cm soils was smaller than that in 30-60 cm soils under SWI treatment. Previous studies have found that the *Comammox Nitrospira* strain can oxidize ammonium nitrogen to nitrate (Daims et al., 2015). This may be the reason for the smaller ^15^N leaching loss rate of 0-30 cm than 30-60 cm soil under SWI treatment. Therefore, the SWI treatment could enhance the NUE of paddy by increasing the relative abundance of microbial communities such as *Bacillus*, *Clostridium*, and *Anaeromyxobacter*.

### 4.5 Effects of different fertilization measures on microbial functional gene abundance

Ammonia-oxidizing archaea (AOA) in the paddy soil may exert a greater effect on soil ammonia oxidation than that exerted by AOB. It was found that the number of AOA in the soil could be up to 3000 times that of AOB (Leininger et al., 2006; He et al., 2007; Shen et al., 2008). This indicated that AOA was the dominant ammonia-oxidizing microorganism in the soil compared to AOB. The relative abundance of AOA and AOB in the SWI groups was higher compared to that in the TI groups during the growth and development of paddy. Additionally, SWI measures were found to increase the NO_3_^-^-N content in both the soil and leaching water (Sections 3.1 and 3.2). The increase of AOA and AOB in the SWI groups promoted the soil nitrification process, thereby increasing NO_3_^-^-N content. The abundances of AOA and AOB during the middle and later stages of paddy planting were higher compared to those during the early stage. This indicated that nitrification is stronger during the middle and late stages of paddy planting, and that NH_4_^+^-N converted by urea was more easily converted into NO_3_^-^-N. The relative abundance of the *nosZ* gene (Equation 12) was lower than that of the *nirS* gene, which may lead to the activity of NO_2_^−^ reduction to NO, which was higher than that of N_2_O reduction to N_2_.

### 4.6 Fate of ^15^N in the soil-plant-water system and environmental implication

In this study, the amount of nitrogen fertilizers applied to plants, soil, and leaching loss in the paddy field system was tracked by adding ^15^N-labeled urea. **Figure 8** shows the fate of ^15^N in the soil-plant-water system after fertilization. It was found that plant absorption and soil residue were the main pathways of the nitrogen fertilizers. The same results were described in Section 3.4. The SWI measures increased the relative abundance of *Firmicutes* in the paddy soil, thereby increasing the ability of nitrogen fixation to produce ammonium nitrogen. Simultaneously, the relative abundances of *Bacillus*, *Clostridium*, and *Anaeromyxobacter* in the paddy soil were increased by SWI measures. *Bacillus* could improve heterotrophic nitrification activity under aerobic conditions (Mevel and Prieur, 2000). *Clostridium* and *Anaeromyxobacter* could promote nitrogen fixation under anaerobic conditions (Masuda et al., 2020; Han et al., 2021). This indicated that the SWI measures could effectively increase the NUE of paddy. Nutrients dissolved in water flow into groundwater as the paddy field is vertically leached. The SWI treatment reduced the amount of leaching water by reducing the amount of irrigation water while ensuring the yield, thereby reducing the loss of nitrogen. This improved the NUE and reduced the risk of non-point source pollution.

During the middle and late stages of rice growth, the SWI groups resulted in higher abundance of AOA and AOB. Therefore, the nitrification was more intense in the SWI group. At this time, the ammonium nitrogen converted from urea is more easily converted to nitrate nitrogen through nitrification. The nitrogen leaching loss in paddy fields is mainly caused by ammonium nitrogen (**Figure 7**). Thus, the SWI measures can effectively reduce the nitrogen leaching loss in paddy fields by controlling ammonium nitrogen.

## 5 Conclusions

The main conclusions of this paper are as follows:

- The SWI treatment promoted the absorption of nitrogen fertilizer by rice through increasing the NO_3_^-^-N content in red soil and inhibiting the migration of nitrogen. Further experiments revealed that SWI treatment increased the relative abundance of *Firmicutes* in the paddy red soil, which were found to be capable of fixing nitrogen and producing ammonium nitrogen during rice growth. This may complement the NH_4_^+^-N content for paddy under SWI treatment.
- In South China with red soil as the main soil type, the SWI treatment could reduce the leaching loss rate of nitrogen fertilizer in the deep soil by 0.30-0.80% and improve the NUE by 2.18-4.43%. Nitrogen fertilizer from SWI treatment tend to accumulate in the surface layer (0-30 cm) of the soil. Plant absorption and nitrogen fertilizer residue were the main pathways of nitrogen fertilizer. After applying the ^15^N-labeled nitrogen fertilizer to the paddy soil, plant absorption accounted for 19.28-28.50% of the nitrogen fertilizer, whereas the leaching loss of each form of the nitrogen fertilizer only accounted for 1.31-4.63%.
- The SWI measure can enhance nitrification and promote nitrate nitrogen accumulation and ammonium nitrogen transformation in red soil. The SWI measure and nitrogen fertilizer application increased the relative abundance of *nirS* and *nosZ* genes and promoted denitrification in the surface red soil of paddy. The SWI measures promoted ammonia oxidation and denitrification through the promotion of *Proteobacteria*, *Nitrospirae*, and *Bacteroidetes* abundance and activity. Compared to AOB, AOA in the paddy soil might have a greater effect on soil ammonia oxidation.

The outcomes from this study are expected to advance the understanding the nitrogen transformation and microbial regulation mechanisms in paddy field systems under different water and fertilizer management conditions. However, limitations still exist in (i) the lack of studying gaseous nitrogen loss in paddy fields; (ii) the absence of data to deeper analysis that the soil moisture content is consistent with the same treatment. Therefore, further studies are needed to quantify the gaseous loss and fate of nitrogen fertilizers throughout the paddy system. At the same time, soil-water potential data and water depth data throughout the crop-growing season need to be analyzed to eliminate the influence of soil moisture content on different irrigational measures.

## Data availability statement

The 16S raw data were deposited in the National Center for Biotechnology Information (NCBI) Sequence Reads Archive (SRA) (accession number: PRJNA1087108).

## Author contributions

TYC: Data curation, Formal analysis, Writing – original draft, Writing – review & editing. XMY: Data curation, Investigation, Visualization, Writing – review & editing. ZZu: Data curation, Investigation, Visualization, Writing – review & editing. HJX: Writing – review & editing, Investigation. XJY: Investigation, Writing – review & editing. XJZ: Investigation, Writing – review & editing. SRH: Investigation, Writing – review & editing. XW: Investigation, Writing – review & editing. XML: Investigation, Writing – review & editing. YTL: Conceptualization, Funding acquisition, Methodology, Resources, Supervision, Writing – review & editing. ZZh: Conceptualization, Funding acquisition, Methodology, Resources, Supervision, Writing – review & editing.
