# Supplementary material for: Shallow wet irrigation reduces nitrogen leaching loss rate in paddy fields by microbial regulation and lowers rate of downward migration of leaching water: a 15N-tracer study
Source: Front Plant Sci. 2024 Mar 25;15:1340336. doi: 10.3389/fpls.2024.1340336 (PMC10999577; doi:10.3389/fpls.2024.1340336)
Supplement: Supplementary file 1 [file DataSheet_1.docx]

**Supplementary Data**

**Shallow wet irrigation reduces nitrogen leaching loss rate** **in paddy fields by microbial regulation and lowers rate of downward migration of leaching water: A ^15^N-tracer study**

**Tianyi Chen^a^, Xiaoming Yang^a,1^, Zheng Zuo^a^, Huijuan Xu^a^, Xingjian Yang^a^, Xiangjian Zheng^a^, Shuran He^b^, Xin Wu^a^, Xueming Lin^a^, Yongtao Li^a,*^, Zhen Zhang^a,*^**

^a^ College of Natural Resources and Environment, Joint Institute for Environmental Research & Education, South China Agricultural University, Guangzhou 510642, China.

^b^ College of Resources and Environment, Yunnan Agricultural University, Kunming 650201, China.

*Corresponding authors:

Zhen Zhang (Email: zzhangal@scau.edu.cn, [zhangzhen_23102@163.com](mailto:zhangzhen_23102@163.com))

Yongtao Li (Email: yongtao@scau.edu.cn).

^1^ The co-first author contributed equally to this study.

*
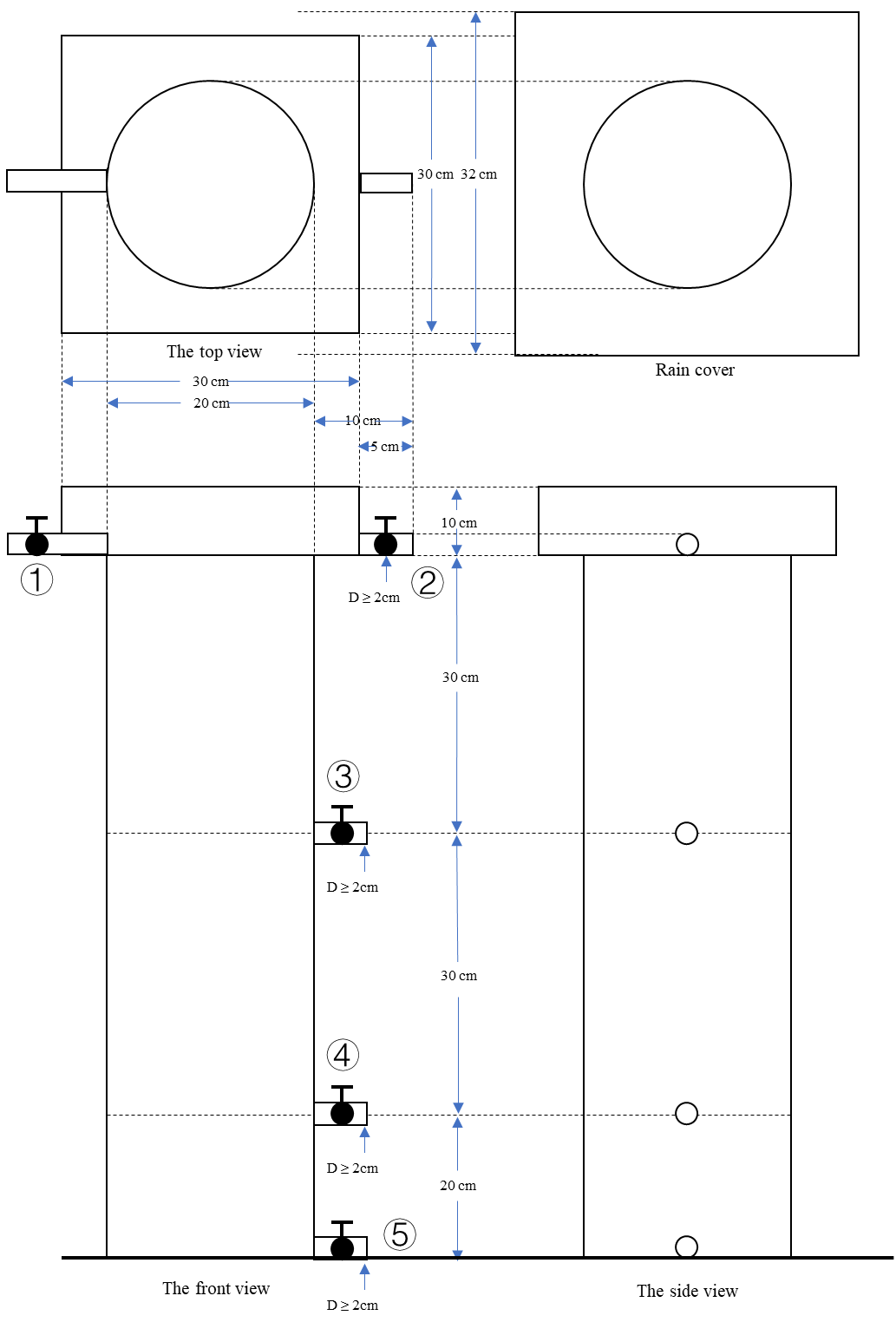
*

**Fig.S.1** Drawing of the design of the test setup.

**Table S.1** Soil and water sample testing indicators and methods

| Sample category | Indicators | Method | Sampling and preservation |
| --- | --- | --- | --- |
| Leaching water sample | TN | continuous-flow analyzer | Opened the ball valve, used a soil leaching sampler to collect soil leaching water at 30 and 60 cm, divided it into centrifuge tubes, acidified it with 1-2 drops of sulfuric acid, and stored it at 4 ℃. |
|  | NH_4_^+^-N | continuous-flow analyzer |  |
|  | NO_3_^-^-N | continuous-flow analyzer |  |
|  | Volume | Measuring cylinder |  |
| Soil sample | Soil bulk density | Ring knife sampling method | Evenly divided the surface soil into 3 points and placed them in plastic bags. Cut opened the bags and let them air dry naturally. Ground individual samples in an agate mortar and divided them into three particle sizes of 2 mm, 1mm, and 0.15 mm. |
|  | alkali-hydrolyzed nitrogen | Alkali diffusion method |  |
|  | TN | Kjeldahl method |  |
|  | C/N | Elemental analyzer |  |
|  | ^15^N abundance | Stable isotope mass spectrometer |  |
|  | NH_4_^+^-N | continuous-flow analyzer | Fresh soil stored in the 4 ℃ refrigerator. |
|  | NO_3_^-^-N | continuous-flow analyzer |  |
|  | Functional genes | qPCR | Fresh soil stored in the -80 ℃ refrigerator. |
|  | Microbial Communities | High-throughput sequencing |  |
| Plant sample | ^15^N abundance | Stable isotope mass spectrometer | Rice was dried at 70°C and then ground and bagged |
|  | TN | Kjeldahl method |  |

**Table S.2** Quantitative PCR primer information

| Gene Name | Primer name | Primer sequences (5′-3′) | Product size |
| --- | --- | --- | --- |
| *amoA*  -Archaea | AOA-F  AOA-R | STAATGGTCTGGCTTAGACG  GCGGCCATCCATCTGTATGT | 638  638 |
| *amoA*  -Bacterial | AOB-F  AOB-R | GGAGRAAAGCAGGGGATCG  CTAGCYTTGTAGTTTCAAACGC | 468  468 |
| *nirS* | *nirS*-F  *nirS*-R | GTSAACGTSAAGGARACSGG  GASTTCGGRTGSGTCTTGA | 413  413 |
| *nosZ* | *nosZ*-F  *nosZ*-F | CGYTGTTCMTCGACAGCCAG  CGSACCTTSTTGCCSTYGCG | 430  430 |
| Bacterial 16S | Bacterial-16S-F  Bacterial-16S-R | CCTACGGGAGGCAGCAG  TTACCGCGGCTGCTGGCAC | 196  196 |
| Archaeal 16S | 16S-rRNA-F  16S-rRNA-R | GYGCASCAGKCGMGAAW  GGACTACVSGGGTATCTAAT | 462  462 |

**Table S.3** PCR amplification system conditions

| Amplification procedure |  | Component | Volume |
| --- | --- | --- | --- |
| 95℃ 30min  95℃ 10s |  | 2xqPCRmix  F primer (10 pmol/μL) | 10 μL  0.5 μL |
| 52-65℃ 30s*  72℃ 30s | 40 cycles | R primer (10 pmol/μL)  DNA templates | 0.5 μL  2 μL |
| 95℃ 15s |  | ddH_2_O | 7 μL |
| 60℃ 60s  95℃ 15s | Monitor every 0.3℃ of temperature rise | Total | 20 μL |

Band * for gradient amplification, gradient temperature is: 52℃、55℃、60℃、65℃.

The DNA template uses a proportional mixture of 5 samples.


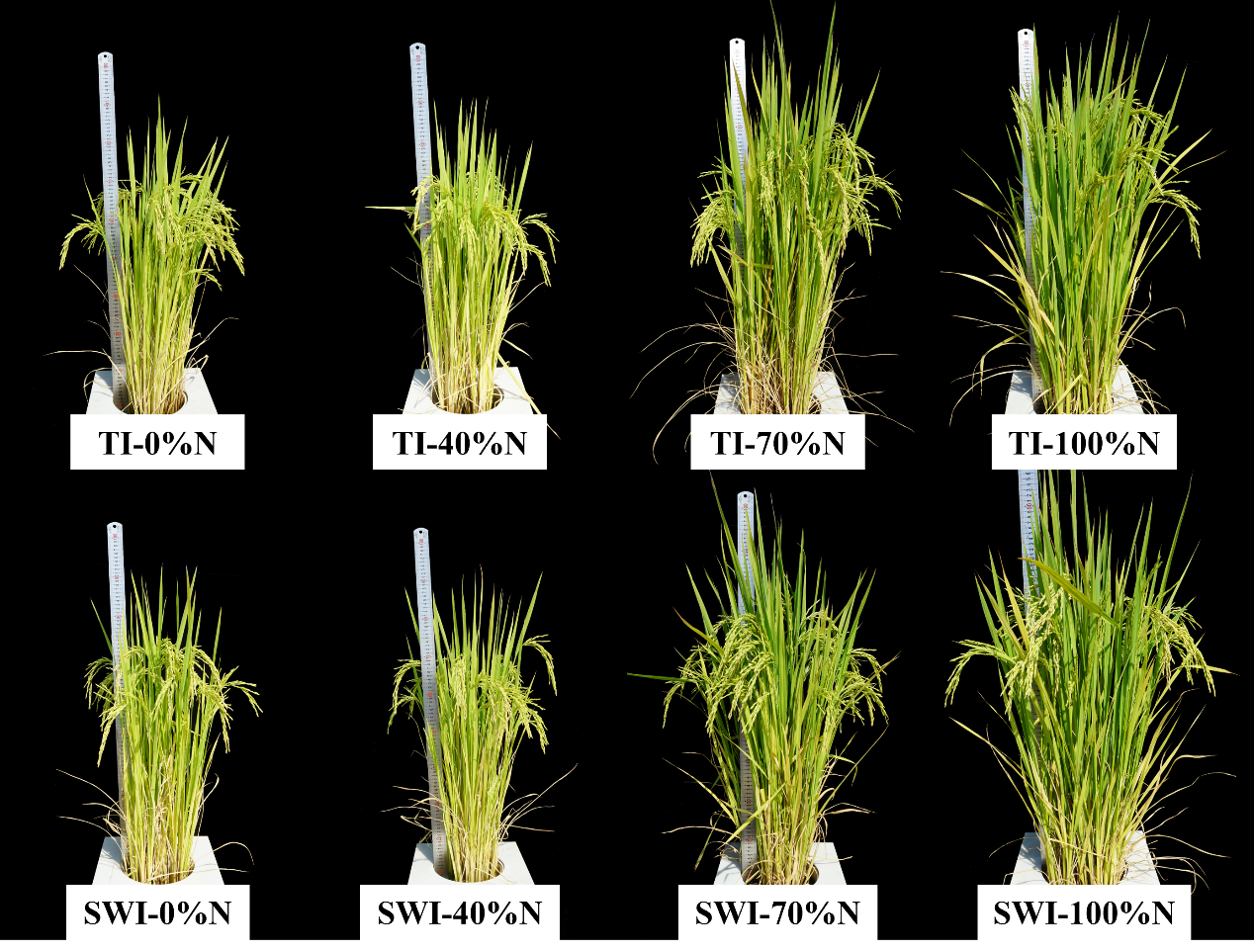


**Fig. S.2** Growth differences of rice in different treatment groups

**Fig. S.3** The amount of rice leaching loss under different treatments


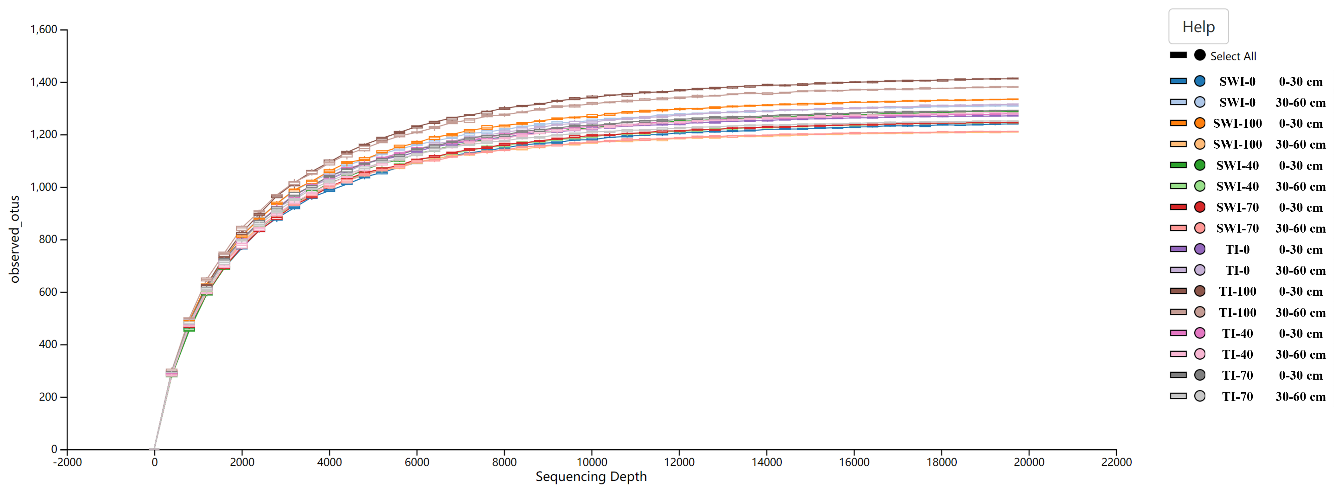


**Fig. S.4** Dilution curves of Alpha diversity index for each treatment group

**Table S.4** Diversity index analysis of soil bacterial communities in different treatments

| Treatments | Chao1 | ACE | Shannon | Simpson |
| --- | --- | --- | --- | --- |
| SWI-0 0-30 cm | 1285.98±133.55a | 1284.5±133.4a | 9.44±0.13a | 0.997±0.001a |
| SWI-0 30-60 cm | 1336.56±129.22a | 1335.17±129.34a | 9.67±0.11a | 0.998±0.001a |
| SWI-40 0-30 cm | 1269.84±76.58a | 1268.33±76.57a | 9.36±0.16a | 0.996±0.001a |
| SWI-40 30-60 cm | 1309.47±102.74a | 1308.5±103a | 9.47±0.22a | 0.995±0.004a |
| SWI-70 0-30 cm | 1260.11±116.61a | 1259±116.47a | 9.47±0.11a | 0.997±0.001a |
| SWI-70 30-60 cm | 1295.4±166.65a | 1294±166.04a | 9.56±0.17a | 0.997±0.001a |
| SWI-100 0-30 cm | 1360.36±151.26a | 1359.33±150.76a | 9.62±0.21a | 0.997±0.001a |
| SWI-100 30-60 cm | 1310.55±201.95a | 1308.6±200.23a | 9.56±0.27a | 0.997±0.001a |
| TI-0 0-30 cm | 1311.37±79.33a | 1309.5±78.87a | 9.56±0.09a | 0.997±0.001a |
| TI-0 30-60 cm | 1307.64±99.27a | 1305.33±98.41a | 9.58±0.14a | 0.998±0.001a |
| TI-40 0-30 cm | 1285.63±139.58a | 1284.33±139.82a | 9.53±0.21a | 0.997±0.001a |
| TI-40 30-60 cm | 1272.56±135.88a | 1271.17±134.81a | 9.39±0.27a | 0.996±0.002a |
| TI-70 0-30 cm | 1344.18±123.05a | 1342.17±124.3a | 9.47±0.37a | 0.996±0.005a |
| TI-70 30-60 cm | 1267.09±119.79a | 1265.5±118.98a | 9.55±0.18a | 0.997±0.001a |
| TI-100 0-30 cm | 1428.13±119.38a | 1368.67±168.44a | 9.54±0.21a | 0.997±0.001a |
| TI-100 30-60 cm | 1379.26±137.95a | 1377.67±136.96a | 9.72±0.18a | 0.998±0.001a |
